# Is it possible to change of the duration of consolidation period in the distraction osteogenesis with the repetition of extracorporeal shock waves?

**DOI:** 10.4317/medoral.21556

**Published:** 2017-02-04

**Authors:** Mehmet-Emin Onger, Cihan Bereket, Ismail Sener, Nilufer Ozkan, Erman Senel, Ahmet-Veysel Polat

**Affiliations:** 1 PhD. Asst. prof. Histology and Embryology, School of Medicine, Ondokuz Mayıs University, Samsun, Turkey; 2 Dent. Assoc. prof. Oral and Maxillofacial Surgery, School of Dentistry, Ondokuz Mayıs University, Samsun, Turkey; 3 Dent. Asst. prof. Oral and Maxillofacial Surgery, School of Dentistry, Pamukkale University, Denizli, Turkey; 4 MD. Assoc. prof. Radiology, School of Medicine, Ondokuz Mayıs University, Samsun, Turkey

## Abstract

**Background:**

In this study we examined the effects of two different repeated Extracorporeal Shock Waves (ESW) on the consolidation period of the distraction osteogenesis (DO) of the rabbit mandible using stereological, radiological and immunohistochemical methods.

**Material and Methods:**

DO was performed unilaterally in the mandible of 18 New Zealand rabbits (six months old, weighing between 2.5-3 kg). In the consolidation period, rabbits were divided into three groups randomly after the distraction period. The distraction zone of the mandible was received no treatment as controls (E0*2). Group 2 (E 500*2) received ESWT (twice 500 impulses at 14 kV and 0.19 mJ/mm2 energy) in the first and fourth days of the consolidation. Group 3 (E1000*2) treated with ESWT (twice 1000 impulses at 14 kV and 0.19 mJ/mm2 energy) in the first and fourth days of the consolidation period. After the sacrification, radiologically bone mineral density, new bone formation, new fibrous tissue and new vessel formation were analyzed by stereological.

**Results:**

It was found a statistically significant difference between the study groups and control group in the bone mineral density measurements and the highest value was in the E1000*2 group. In the stereological analysis, new bone formation was highest in the E1000*2 group and there was a significant difference compared to the other groups (E0*2 and E500*2) (*p*=0.000). The lowest connective tissue volume was found in the E500*2 and there was a significant difference compared to the other groups (E0*2 and E1000*2) (*p*=0.000). The volume of the new vessel was highest in the E500*2 and lowest in the E0*2 group. It was found statistically significant difference between the values of the study and control groups.

**Conclusions:**

Interestingly, we found that repetition of the 1000 impulses ESWT accelerated the consolidation, 500 impulses ESWT extended consolidation period of the DO.

**Key words:**Distraction osteogenesis, extracorporeal shock waves, stereology, rabbit.

## Introduction

Distraction osteogenesis (DO) is a biological process characterized by new bone formation parallel to the distraction vector as a result of the induction of callus by application of tensile strength to the bone segments divided by the methods of corticotomy or osteotomy (1). The DO technique is a popular method that is being used in the treatment of the congenital or acquired cranio-maxillofacial defects (2). One of the major disadvantages of the technique is the long treatment period, which is associated with the consolidation phase that takes place in 8 to 12 weeks (3). Therefore, it is claimed that the acceleration of the regeneration by the way of stimulation of the callus in the process of consolidation would shorten the time for treatment. It is focused on the studies aimed to increase the mineral density, quality of content and resistance of the newly formed bone (4,5). Low intensity ultrasound, recombinant growth factor, intermittent parathyroid hormone, calcitonin, alendronate, calcium sulphate, bone morphogenetic proteins (BMP), transforming growth factor (TGF), vascular endothelial growth factor are used in DO in order to determine possible effects of them which improve the bone healing and shortening the consolidation period (5-7).

Extracorporeal Shock Wave Treatment (ESWT) is a conservative method used in the treatment of the muscular and skeletal deformities such as unfused fractures, lateral epicondylitis, plantar fasciitis and calcified tendinitis (8,9). It is known that ESWT enhances the healing of the fractures in long bones by increasing the differentiation and vascularization. Authors, in their previous studies, showed that ESWT increases the amount and biomechanical properties of the new bone (9,10). ESWT has become a technique that is acknowledged worldwide. It is reported in the literature that it is used successfully in the treatment of several diseases. In spite of these developments, the exact mechanism in which the shock waves exert their positive activity has not been understood yet (11). It was shown among the advantages of ESWT, that it is a conservative method, it has a low rate of complication, its effectiveness in the indications in which the other methods are not, its low cost (11). It advised that it is used in treatment of complications like delayed fusing and inability to fuse (12).

Under the light of these data, the aim of our study is to investigate the effects of different doses of ESWT on fracture healing using unbiased stereological techniques quantitatively in addition to radiological approach.

## Material and Methods

18 New Zealand rabbits, 6 months of age and 2.5-3 kg of weight were used. The animals were collected from Experimental Animals Research and Applying Center of Ondokuz Mayıs University and were randomly divided into three groups as one control, and two experimental groups. The study was supported by the foundation of scientific research projects of the Ondokuz Mayıs University.

All surgical procedures and experimental design including distraction processes were performed according to a previous study (13). This study was approved by the Ondokuz Mayıs University Animal Experiments Local Ethical Committee, in 25/11/2011, with the number of 2011/66.

- ESWT applications

After the latent period of 5 days, the mandibles of the animals were distracted with a 0.35 mm/12 hour distraction rate, for 10 days. Then, the animals were divided into 3 random groups (2 experimental, 1 control). The experiment design was as follows: To the 1st group (CONTROL, n=6) ESWT was not applied. To 2nd group (E500*2, n=6), twice doses of 500 impulses ESWs with 14Kv (equivalent to 0.19 mJ/mm2) were applied using an ESW device (OE050 focused applicator, Orthogold 100, MTS, Konstanz, Germany). To 3rd group (E1000*2, n=6), twice doses of 1000 impulses were applied using same device. ESWT was applied on the 1st and 4th days of the consolidation period to the distraction zone. ESWs were performed after the application of surgical lubricant gel to the skin. At the end of the 28 days of consolidation period, the rabbits were sacrificed with high dose sodium pentobarbital (Pental; IE Ulagay, Istanbul, Turkey).

- Stereological evaluation

**Figure 1:**
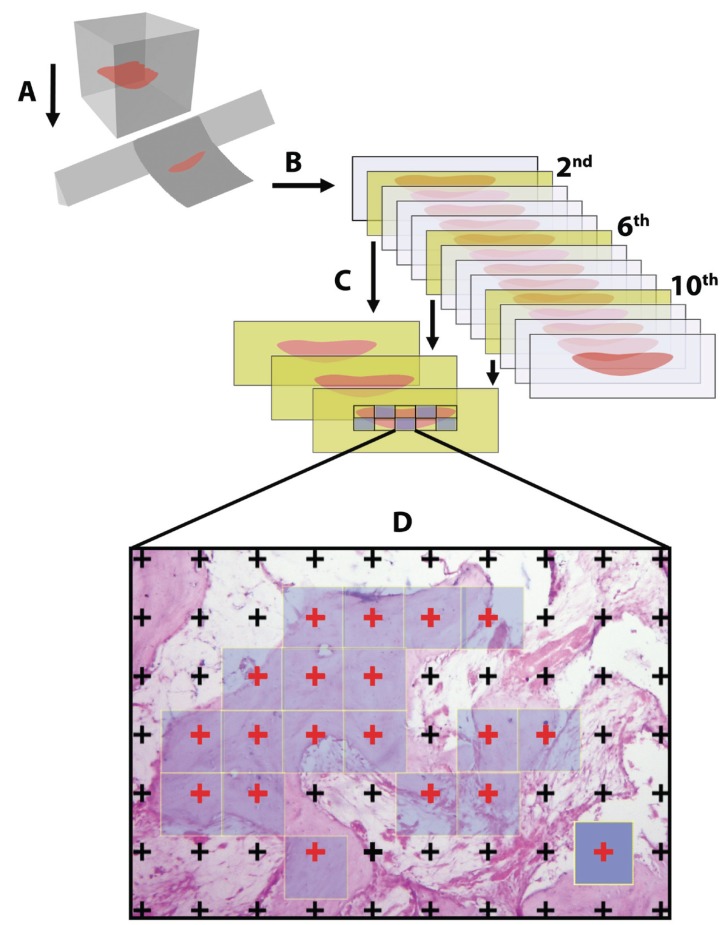
Schematic illustration of stereological steps indicates sectioning (A and B), selecting of the sections according to the systematic random sampling manner (C), and the estimation of the related parameters on selected areas (D).

**Figure 2:**
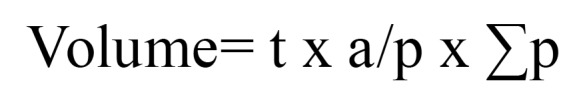
Formulae

The stereological evaluation of the tissue samples was performed in Ondokuz Mayıs University Faculty of Medicine, Histology and Embryology Department laboratory. The tissues were decalcified in Formic acid (5%) for 21 days. After routine histological processing, serial sections (7-μm thickness) were taken (Fig. 1A). According to the systematic random sampling manner, which is the most unbiased way to select sections stereological, sections were selected (Fig. 1B,C). Then selected sections were stained with Hematoxylin-Eosin (HE) and photographed by using light microscopy (Leica M 4000 B, Germany), with color digital camera (Microbrightfield, Williston, VT, USA) in stereological analysis system (Stereoinvestigator 9.0, Microbrightfield, Williston, VT, USA). The Cavalieri method was applied to light microscopy images in order to estimate the total volume of new bone, connective tissue infiltration and capillaries (14,15). Point-counting grids were used in determination of area in sections (Fig. 1D) . The point density on point-counting grids was determined to have an appropriate coefficient of error (CE) for the distracted area in the slides after performing the pilot study. The coefficient of error and the coefficient of variation were determined in accordance with the formulae (16) (Fig. 2)

(‘t’, section thickness; ‘a/p’, the area representing the each point on the point-counting grid (Dark blue square); ‘∑p’, the total number of points that hit to the interested area (Red points)) (Fig. 1D).

- Immunostaining Procedures

The immunohistochemical staining of sections also was performed on sections using BMP7, TGFβ1, VEGF, and collagen antibody.

- Radiologic Evaluation

Following a consolidation period CT images were taken in all groups. For this purpose; all rabbits were positioned with the occlusal plane perpendicular to the horizontal plane. Images were taken by HR-CT (Aquilion 16 system, Toshiba Medical System Corporation, Tochigi-ken, Japan) according to (17). Bone density values were measured twice by the same examiner.

- Statistical Analysis

The data obtained from densitometric and stereological evaluations were compared with one-way ANOVA using SPSS (Ver: 21, IBM Corporation for Mac, USA) statistics program. The p values less than 0.05 were accepted as significant.

## Results

- Radiological Findings

**Figure 3:**
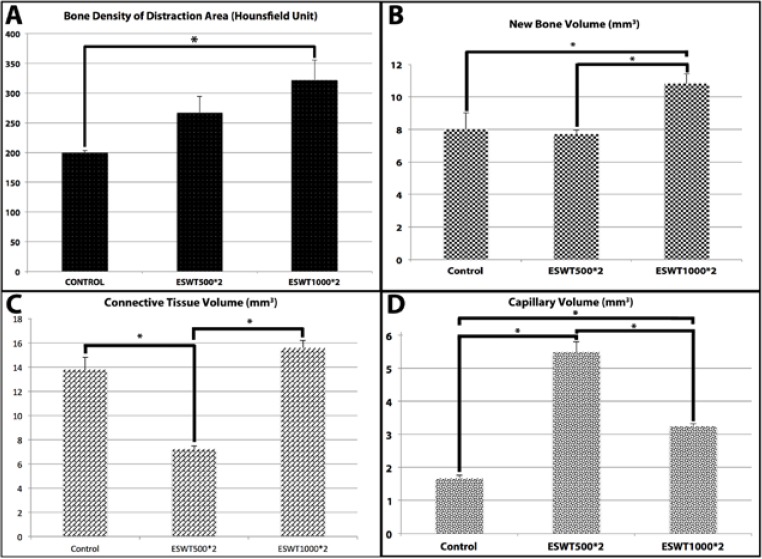
Bone density (A), new bone volumes (B), connective tissue volumes (C), and capillary volumes (D) of the groups in the distraction areas.

When the results obtained from the measurements of the bone density (Hounsfield Unit) of the distracted area in Computed Tomography, as the bone density of all the experimental groups were found to be higher than the control group (Fig. 3A). The highest values were ESWT1000*2 and ESWT500*2, in decreasing order. While there was no difference between ESWT500*2 and the control group, there was significant difference between ESWT1000*2 and the control group.

- Stereological findings

**Figure 4:**
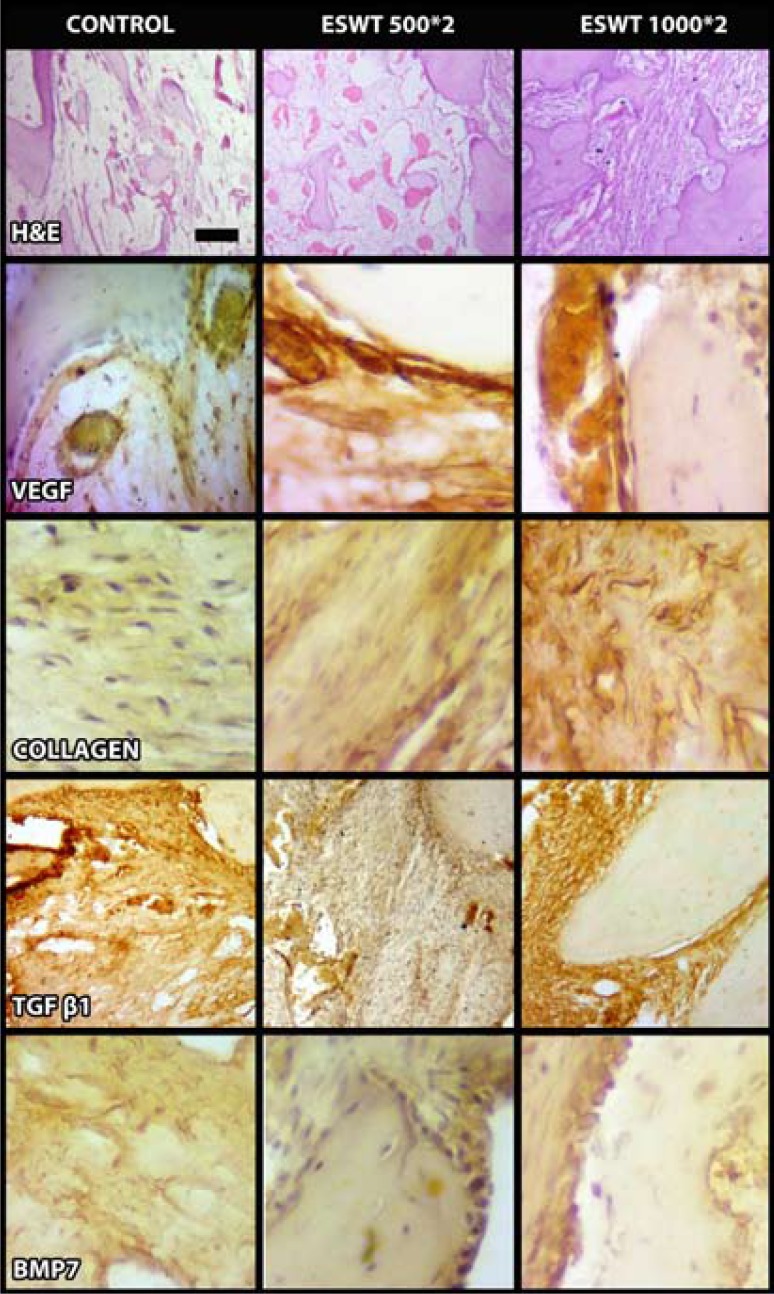
Representative histological images of the groups including immunohistochemistry.

New bone volume: There was no difference between ESWT500*2 and control groups (*p*>0.05), whereas there were significant differences between the groups ESWT1000*2 and control, ESWT1000*2 and ESWT500*2 (*p*<0.00) (Figs. 3B, 4).

- The connective tissue volume: There were statistical differences between the groups ESWT500*2 and ESWT1000*2, and ESWT500*2 and control (*p*<0.00), while there was no difference between ESWT1000*2 and control groups (*p*>0.05) (Figs.3C, 4).

- Volume of capillaries: Statistically significant difference was found between the all groups in point of capillary volume (*p*<0.00) (Figs. 3D, 4).

- Immunohistochemical Findings

The immunohistochemical staining also have supported the other results in the groups. The more positive areas of staining with VEGF, collagen antibody, BMP7 were found in the ESWT1000*2 group (Fig. 4).

## Discussion

DO is a biological process in which the osteotomized bone tips are separated gradually from each other by applying tensile strength and the consequent new bone formation occurs. This method, which enables the soft tissues to accommodate the lengthening as well, becomes a popular technique recently and the usage of this technique in craniofacial area opened up new possibilities for the treatment of the patients with maxillofacial anomalies and deficiencies (18).

The deformities in the maxillofacial anomalies can be reconstructed with conventional orthognathic surgery procedures although these procedures might cause postoperative complications (19,20). One of the most significant problems in orthognathic surgery is the adaptation of the soft tissue. In some cases, the soft tissue that enclose the bone may not adapt sufficiently to the new position because of the movements and this situation may lead to aesthetic problems (21). The complications of the orthognathic surgery include donor field morbidity, relapse risk and inability to perform extensive skeletal movements (22). DO technique is used in the treatment of the maxillofacial deformities to prevent and minimalize these problems (23).

Clinicians carried out various experimental studies about the shortening of the consolidation process by speeding up the new bone maturation in DO, in order to prevent these problems. These include; bisphosphonates, thrombocyte-rich plasma, hormones, demineralized bone matrix, calcium sulphate, electrophysiological applications, low-intensity laser, growth factors, shock waves, ultrasound, hyperbaric oxygen (HBO), bone grafts, cytokines, stem cells (24).

ESWT is a non-invasive method that is used in the treatment of unfused fractures, lateral epicondylitis, plantar fasciitis, calcified tendinitis, muscle and skeletal deformities (9,25). It is reported in the literature that ESWT accelerates the healing in long bones by increasing the cell differentiation and neovascularization, in animal models. Authors, in their studies on rabbits, reported that ESWT increases the callus amount and biomechanical properties (9). However, there is no sufficient studies on the effects of ESWT on the new bone formation in DO (26).

In a study (9) the biological effects of ESWT on bone healing in rabbit tibia fracture model was investigated. They stated that, in the experimental group, the new bone durability was higher, more cortical bone formation and neovascularization was observed, the osteogenic and angiogenic growth factors such as VEGF, endothelial nitric oxide synthetase (eNOS), proliferating cell nuclear antigen (PCNA) and BMP-2 were secreted more. Again, in another study (27) in which the effects of ESWT on alveolar bone regeneration in rats was investigated, it was reported that the alveolar bone regeneration was higher in rats on which 300 and 1000 shot ESWT was applied and that this effect continued for 6 weeks. It was also reported by another group of researchers (26) that ESWT is effective in increasing the angiogenesis and bone regeneration and in shortening the consolidation process.

In our study, the possible positive effects of different doses of ESWT on regeneration in distraction osteogenesis model were investigated. The double 500- and 1000- shot doses of ESWT were compared with control group. It is revealed that all the different application types of ESWT have positive effects on distraction osteogenesis. This result was supported by the Hounsfield unit values measured by CT. Among the groups, the most effective dose was found as ESWT 1000 *2.

When the effects of the different doses of ESWT on new bone formation was investigated of stereological evaluation, the low dose of ESWT was found no beneficial effect on improving the new bone volume. The fact that the new bone volumes are high in ESWT 1000*2 group is supportive of its positive effects on regeneration. These findings of ours are consistent with the results of a previous study (26). There is no chance of comparing our study with other studies because of the variability of the ESWT’s energy density applied to the bone regeneration, the shot counts, the shock wave production type and the devices. General findings show the positive effects of ESWT (9,26,27). Because that ESWT is a relatively new treatment, further studies in this area are required to determine the optimum doses.

In this study, ESWT’s different treatment protocols on DO were tested. ESWT 1000*2 application became more prominent. Our results were supported by the results of immunohistochemical staining. We are in the opinion that this dose can be used in shortening the consolidation process. New experimental studies are required to comprehend it better. ESWT’s positive contribution to bone quality and mineralization by its usage in consolidation process might be beneficial in preventing the possible complications that might occur in the long treatment period of DO. Nevertheless, this situation requires being enlightened sufficiently with experimental studies.
